# Lectures on the Comparative Anatomy and Physiology of the Invertebrate Animals, &c.

**Published:** 1844-01-01

**Authors:** 


### Medico-Chirurgical Review,

### No. LXXIX.

[No. 39 of a Decennial Series.]

OCTOBER 1, 1843, TO JANUARY 1, 1844.

I. Lectures on the Comparative Anatomy and Physiology of the Invertebrate Animals, &c. By *Richard Owen*, F.R.S. 8vo. pp. 392. Longman and Co. 1843.

II. Elements of Anatomy. By *Jones Quain*, M.D. Fifth Edition. Edited by Professors *Richard Quain* and *William Sharpey*. 8vo. pp. 424. Taylor and Walton, 1843.

III. The Physiological Anatomy and Physiology of Man. By *R. B. Todd*, F.R.S. and *Wm. Boroman*, F.R.S. &c. Part I. 8vo. pp. 200. Parker, 1843.

IV. Popular Cyclopædia of Natural Science; Article, Animal Physiology. By *Wm. Carpenter*, M.D. &c. 12mo. pp. 579. Orr and Co. 1843.

Unquestionably one of the greatest discoveries, that has ever been atchieved in physiological science, is the doctrine of what has been called Cell-Formation. By this term we mean to imply that all living bodies and all organic structures are primarily and essentially composed of simple cells or vesicles; these cells or vesicles containing the germs within themselves of other and new cells, in every respect like to those from which they sprung. The subject is one not only of the highest interest to the student of natural science, and to every one who takes an interest in searching into the mysteries of Creative Wisdom, but is of direct importance to the scientific physician, as affording a means of explaining not a few of the most curious phenomena exhibited by living bodies, in a state of disease as well as of health. Indeed it cannot be otherwise. Whatever advance is made in physiology, must inevitably lead to more enlarged and correct views on pathological inquiries. He, who understands the living machine the best, ought surely to be the most skilful in assisting Nature to heal its injuries and compose its disorders. All our wisest practitioners have been, in their day, keen inquirers after the discoveries of physiology; and many of the most valuable therapeutic suggestions have been derived from the skilful application of these discoveries to practice. Need we say more to engage our readers' attention to the subject which we have selected for our present remarks ?

The field of inquiry, which has been thus opened up, is most extensive and full of the most curious details; for the entire productions of the Vegetable, as well as of the Animal, world are embraced within its grasp. The law or principle of Cell-formation is applicable to all living things without exception—from the oak of the forest to the lichen on its branches, from man himself to the microscopic animalcule that eludes his unaided sight.

It is not possible for any one yet to appreciate the full magnitude and value of the discovery in question, or to estimate all its bearings on physiological and pathological science. Already it has given an entirely new aspect to the one, and it has served to explain some of the most remarkable phenomena in the other; while it still continues to bestow a rich harvest of reward on every patient observer of living Nature. But it may be here asked, to whom are we indebted for this important discovery? It is to Germany that we owe the boon. She may well be proud of her *Schleidens*, and *Schwanns*, and *Müllers*, and *Purkinjes*—not to mention others—for to them belongs the undivided honour of the atchievement. With such labourers as these in the vineyard of Physiology, and with a *Liebig* in that of Chemistry, Germany may justly claim to herself, just now, the foremost place of honour in two of the most attractive departments of physical science.

Truly the more that we know of Nature's works, the more are we lost in wonder at the magnitude and variety of their details, contrasted with the fewness and simplicity of the elements or agencies that are employed to produce them. Many are the proofs of this truth, afforded by the operations of the material Inorganic world around us. The spark, elicited by rubbing a glass rod in our hands, is the offspring of the same cause that produces the lightning flash; the spurting crack of a piece of new-lit coal is but a parlour experiment, so to speak, that shews the terrible agency of pent-up gaseous matter, when it bursts forth in the volcano and the earthquake; and who is there that does not know that the very same law, that brings a tossed-up pebble to the ground, presides over and directs all the motions of the heavenly spheres? No less wonderful are the instances, afforded by Animated nature, in illustration of the same great truth—that grand and ever varied effects are evolved from the simplest causes. Everything indeed reveals a unity of design and a harmony of arrangement, proclaiming with a voice that cannot be mistaken, that there is One great Lord and Architect of all, in whom and by whom Nature lives, and moves, and has its being.

The increasing attention that began to be paid, some twenty or thirty years ago, to the study of Embryology, unquestionably prepared the way for those discoveries of later days, which it is our purpose on the present occasion briefly to explain.

When it was first announced that the human Fœtus, at certain early periods of its intra-uterine life, very closely resembles, in some of its structural arrangements, the configuration of various subordinate families of the zoological series, the assertion was received by many, in this country, as the mere whimsical idea of a transcendental visionary. Few were aware that, more than half a century ago, our own great countryman, *John Hunter*, had distinctly enunciated this most curious and interesting fact. Let us hear what the able illustrator of his works, Mr. *Owen*, says on this subject.

"Some of the medical acquaintances of John Hunter, who, we are told, complacently apostrophised his pursuits, in the language of pity, when they found him dissecting a snail, a bee, or a worm, little dreamt of the expanded views of the animal organisation at which he was obtaining glimpses through those narrow casements. It would seem that Hunter himself was oppressed with the grandeur of the prospect, with the extent of the generalisations which his investigations of the lower animals, and of the embryonic forms of the higher ones, were forcing upon his reflective mind, if we may judge from his struggles to express ideas, at that period so novel and so vast, and to which he could but give imperfect utterance. 'If we were capable,' he says, 'of following the progress of increase of the number of the parts of the most perfect animal, as they first formed in succession, from the very first, to its state of full perfection, we should probably be able to compare it with some one of the incomplete animals themselves, of every order of animals in the creation, being at no stage different from some of those inferior orders; or, in other words, if we were to take a series of animals from the more imperfect to the perfect, we should probably find an imperfect animal corresponding with some stage of the most perfect.'" 367.

In this truly prophetic sentence, the Newton of physiology seems to have clearly anticipated the most important discoveries that have been made, of late years, in the department of Formative and Embryotic Anatomy. The attentive reader cannot fail to remark that, if we carry out the line of reasoning suggested in the paragraph now quoted, it leads us on to the very threshold of the Doctrine of Cell-Formation. For is it not the case that *Hunter* alludes to the embryos of the higher animals being, at different periods of their evolution, (most probably) comparable to the mature and perfect condition of "every order of the lower ones?" If so, the primordial and earliest condition must be that of the lowest of all the animal forms; viz: that of a simple vesicular bag or Cell. How interesting it is to mark the anticipating views of great original minds! As we shall probably revert to this topic hereafter, we need not say more at present.

Without further preface therefore, we proceed to the immediate subject of our present inquiry—viz: to illustrate the doctrine of Cell-formation;—and with this view we propose first to take a rapid review of the general development of the Vegetable and Animal kingdoms, and then to notice the minute structure and mode of evolution of the Human frame.

The simplest form of vegetable life is that of a *cell* or vesicle, containing a fluid in which a number of granules or germinal atoms are observable. This is the structure of that minute aerial Fungus, known by the name of the *Red Snow* or *Gory Dew*, which gives a beautiful vermilion tint to the snow of the Arctic regions. Such, also, is that of another simple Fungus, which becomes developed in *yeast*, and the production of which is intimately connected with the process of fermentation. If a portion of yeast be examined with the microscope, we observe a number of isolated or separate cells, each of which will be found to shoot out from their surface one or more buds. These gradually become cells like the parent one from which they arose; and, ere long, they give origin to others like themselves,—the successive generations being usually arranged in a bead-like or moniliform manner. This process of extension goes on so rapidly that, within the course of a few hours, five or six cells may have become developed in a row from the primary one. Sometimes one of these cells is observed to burst, and to give issue to a number of granules; these are the germs of a new brood of cells. Of a nearly similar nature to the Red Snow and Yeast Plants, seems to be that reddish or green-coloured slimy matter which often forms on cold damp surfaces: it is found to consist of an aggregation of isolated cells or vesicles, in the midst of a semi-fluid substance which holds them together.

The Fungi, to which we give the names of the *Blue-Mould, Mildew, &c.*, and which are so apt to form on decaying animal and vegetable substances, may be considered as exhibiting the first step of advance in organised structure. They consist of jointed filaments which, on examination, are perceived to be composed of very minute elongated vesicles, laid on end to end, or collected together into an aggregated mass. In some of them, each joint, on separating, is capable of forming a new structure like its parent; while, in others, part only of the vesicles seem to possess this power; the fertile ones being placed at the extremities or tops of the rest, which form the stalk of the simple plant. In a still higher tribe, the fertile cellules are collected together within a special envelope.

In the Lycoperdons or puff-balls, the *sporules* or germinal grains are mixed with filamentary shreds and inclosed in a tough membranous envelope; while in the Agarics or proper Mushrooms there is a distinct stem, which separates the reproductive apparatus, situated in the *pileus* or cap, from the nutritive and absorbent apparatus of the root. Yet in them, as in the very simplest kinds of Fungi, the organization is merely and entirely a congeries of distinct but associated cells.

The structure of the *Confervæ* does not differ in any essential respect from that which has been now described: it is nothing but a row of vesicles held together by a common enveloping membrane. Even that of the *Algæ* or proper *Sea-weeds* is scarcely removed from it; the rows of cells, of which they consist, being merely arranged side by side, so as to form their foliacous expansions—which, it is well known, sometimes attain an extraordinary length of development. Nearly the same remark may be made of another tribe of protophytic plants, the *Lichens*, which form so abundantly on old rocks and trees. Their dry and indurated upper surface is formed by the desiccation and consolidation of their primordial cells, (in consequence of constant exposure to the air and the heat of the sun) while their moist surface beneath serves to absorb nourishment, and to reproduce their generative Sporules.

The Characeæ, the Hepaticæ or liver-worts, and the Mosses, may be considered to rank, in point of structural development, immediately above those forms of vegetable life already mentioned. In some of the *Charæ*, the tissue or substance of the plant seems to be almost as simple as that of the *Confervæ;* and it is only, by the greater complexity of their organs of fructification, that they are placed in a higher scale. Some of this family afford a most favourable opportunity to the physiologist of watching the development of the vesicular structure in plants: the new cells or vesicles may often be seen gradually forming at the extremities of the previously existing rows, that constitute the tubular filaments of which a

Chara consists. There is no appearance of true vascular tissue in its structure, although the circulation (as it has been rather improperly called) of granules in the separate joints of the plant might lead a person at first to suppose so. The Hepaticæ, also, consist entirely of mere cellular or vesicular tissue, no distinct tubes or vessels being discoverable in any part of their structure. It is in the *Mosses* that we meet with the first traces (and they are only rudimentary) of a vascular arrangement. These beautiful members of the Cryptogamic family are provided with a fibrous stem, from whose base a number of radicle filaments shoot out, and which bears numerous leaves, regularly arranged upon it. These leaves exhibit the appearance of veins ; but they are not composed, (as in the case of the higher plants,) of woody fibre and vessels, but only of a prolonged form of cellular tissue ; and we find no *stomata* or breathing apertures on their surface, except in a very few instances. Mosses possess a remarkable tenacity of life, and power of resisting injurious influences which would destroy almost all other plants. Hence they often thrive on the most barren surfaces, and in situations where no vegetable forms, except those of the very simplest organization, could exist. The *Ferns* are still more highly organized than the Mosses ; for in them, (when fully developed), not only is there a distinct system of vessels for the circulation of the sap—there are no spiral or air-vessels however—but the appearance of their roots is very similar to that of Phænogamous plants. It is therefore probable that nearly all their nourishment is derived from the soil by their means ; their leaves serving for the aeration or respiratory function of the plant. It deserves however especial notice that *Ferns*, in the early stage of their development, before as yet there is any appearance of stem or leaves, are merely *fronds ;* in every respect like those of the Marchantia, (one of the Hepaticæ or Liver-worts), and which are utterly destitute of any traces of vessels,—their whole tissue being nothing but an aggregation of cellules or vesicles. We may remark, *en passant*, that here we have a beautiful example, in the vegetable world, of that curious law which has been so successfully worked out in animal Embryology, viz : that the more highly organized beings pass through a succession of evolutions or transitory formations, each of which is represented or typified by the permanent condition of some of the lower tribes.

Even in the highest orders of plants, all the vegetable tissues are essentially and primarily vesicular. It is difficult, indeed, to discover any trace of this organisation in the mature condition of some parts, as of the hard woody fibre, bark, &c. ; but, even in these, it is readily perceived, if we examine them in the early stage of their development. In the leaves, flower, and fruit, however, this structure is always abundantly obvious ; more especially in the case of juicy succulent plants.

As a matter of course, we cannot pursue this most interesting enquiry through its minute and varied details ; those, who wish to study the subject as it deserves to be, must have recourse to the works of *Mirbel, Decandolle, Brown, Lindley, Schultze*, &c. on Vegetable Morphology. As the following passage from *Baly's* translation of *Muller's* Treatise on Physiology briefly enunciates the great leading truths to which we have been alluding, we gladly avail ourselves of it.

" The observations of *Mirbel* had shewn that all the forms of vegetable tissue are developed from cells, which at first constitute the whole mass of the tissue, but afterwards undergo various changes in their shape and size, so as to be converted into the woody fibre, spiral vessels, &c. M. *Schleiden* has more recently traced the development of the vegetable tissue at a still earlier stage. The abundant gum of the nascent parts of plants, such as the youngest albumen of a seed, when examined with the microscope, is seen to be turbid from the presence of minute molecules. Soon, larger granules are also observed in it. Around these granules, by a kind of coagulation, larger bodies are formed—the *cytoblasts* or cell-germs—in which the above-mentioned granules are still visible as nuclei. When the germ has attained its full size, a small vesicle appears on it; this enlarges and becomes the cell, in which the germ is still visible, either attached to its wall, or free in its cavity."

Objections have been made to some of the details of *Schleiden's* theory; but this is not the proper place or time to discuss any of the controverted points. It is sufficient for our present purpose to state that it is now universally admitted that the primordial tissue of all plants, from the highest to the lowest, is uniformly and invariably the same.

So much for the external characters and general anatomy of vegetable productions. Let us now, in the prosecution of our subject, proceed to notice briefly the mechanism, so to speak, of two of their most important functions, Nutrition and Reproduction; and we shall find, at every step of our enquiry, numerous and most interesting proofs of the important part which *cells* perform in the operations of living beings.

In such plants as the Red Snow and Yeast Fungi, the process of absorbing the food is performed by every point of their surface alike. There is no distinction of parts; the plant may be said to be all root. But let it be remembered that, when we use such an expression, we enunciate only one of the functions performed; for, besides the act of absorbing nutriment, the simple vegetable cell, that constitutes the entire structure of the lowest vegetables, must have the power not only of assimilating this nutriment with the substance of its body, but also of respiring the air and producing certain changes in its constitution; not to mention another indispensable function, that of generating or reproducing its like. Each and all of these acts are quite as necessary to the humblest form of vegetable life, as to the very highest of the Flowering or Phænogamous plants. How marvellous is the fact! How pregnant with suggestions for deep and generalising reflection!

From the Red Snow and Yeast Fungi, we advance to the Algæ. In their case, there is ample reason to believe that the process of absorbing the food is going on with equal and ceaseless activity, at every point of their surface, by each separate cellule of which their tissue consists. By keeping this simple fact steadily in view, we can readily understand how it is that separate portions of such plants have an independent existence, and we can in some degree explain their extraordinary tenacity of life, not to mention their facility of reproduction.

In the highest of the Fungi, the proper Mushrooms, we find the earliest appearance of rootlets; the stem at the base gives out a number of radicle filaments, that seem to absorb the decaying matter from which these plants derive their nutriment. The sap is then conveyed upwards—not by any distinct vessels or tubes, (for there are none in any part of the vegetable,) but only through the inter-cellular passages—to the top or *pileus*, where it is diffused in every direction; for it is in this part that the germinating granules or *spores* are invariably present.

The Mosses, and allied genera of plants, are always provided with radicle filaments; and sometimes we observe similar appendages to proceed from the sides of the stalks and also from the lower surface of the leaves. But it has been fairly questioned whether these filaments are not intended rather for the support of the plant, than for the absorption of its nutriment. However this may be, all Botanists, we believe, admit that it is chiefly by the surface of their stem and leaves that Mosses derive most of their nourishment. But this is certainly not the case with the members of the highest Cryptogamic family, the Ferns. In them, as in all Flowering or Phænogamous plants, the function of Nutrition is chiefly performed by the *Spongioles* or spongy tips of the rootlets. On examining the structure of these parts, we find that they consist almost entirely of *cells;* a central fasciculus of minute tubes passing up from these to the larger roots, for the transmission of the absorbed fluid.

That, however, an active absorption of nutriment may be carried on by the general surface of certain flowering plants, is obvious from what we know of the growth of many of the Cacti, and of those Orchideæ, which have acquired the name of Air-plants from their growing suspended in the atmosphere. Here, therefore, we have a recurrence, as it were, to the primary organisation of vegetable structure, when every point of the surface was endowed with an equal power of absorption. Whenever, too, an impediment arises to the regular performance of this function by the radicles of a plant, the absorbing activity of the surface of the leaves &c. becomes immediately increased, to compensate for the loss that would otherwise be sustained. Something of the same kind is observed in the case of animals, when any obstruction to the admission of food into the stomach and bowels exists. The lymphatics of the skin will then often act with unusual energy in absorbing fluids; and hence, by the use of baths of milk and such like substances, a considerable quantity of nutriment may occasionally be introduced into the system, for a length of time. This vicarious performance of duty will cease to surprise us, when we bear in mind that (as we shall afterwards shew) the primary organisation of the cutaneous and mucous surfaces is strictly the same; just in the same manner as that of the Spongioles of the roots is identical with that of the Leaves of a plant. But this only in the way of parenthesis.

Proceeding in our enquiry respecting the function of Nutrition in the higher plants, we find that the nutriment, which is absorbed by the roots, rises, as *crude sap*, through a special system of vessels in the stem, and is thence conveyed to the leaves, there to undergo very curious and important changes from the action of the atmosphere on the living juice. The leaves are the Lungs of the plant; it is by them that the function of Vegetable respiration is performed. A quantity of aqueous vapour is exhaled; and at the same time carbon is absorbed from the atmosphere, and becomes assimilated with the sap.

These two important functions—of Exhalation and of the fixation of Carbon—are performed by the *cells*, of which the parenchymatous tissue of leaves consists. It is only after they have been duly performed, that the Sap, on its retrograde course to the stem, is found to have acquired its proper and characteristic properties in each plant: it is then called *elaborated sap, proper juice,* or *latex.* A very remarkable change has taken place in its physical, as well as in its sensible, qualities. On its ascent, it was simple, homogeneous, and generally tasteless; but, on its descent, it is not only more or less sapid and energetic, but is also found to contain numerous cellules or *globules,* which may be seen to move about, even when the sap has been allowed to flow from the plant. These globules may be compared to the globules of the Chyle in animals; and also to the simple elementary cells, that constitute the lowest vegetable forms. In all probability, it is by their agency that the formation of new matter is constantly going on, at every point of the substance of a plant. "According to the observation of *Amici,* the glutinous sap of the vine when removed from the stem assumes, during the species of coagulation which it undergoes, regular forms, closely analogous to those of the lower Confervæ on the one hand, and to the elementary tissues of which it supplies the materials, on the other. When wounds have been made in the course of its flow, it is evidently from the exudation which takes place at their edges that the regeneration of substance takes place; and it scarcely admits of a doubt, therefore, that the ingredients it contains are not *mere* chemical combinations of elementary bodies into organisable products, but that they are to a certain extent possessed of vital properties, which have been probably developed in them simultaneously with the traces of organisation they exhibit."

With this extract we close our remarks on the mechanism (if we may be allowed the phrase) of the function of Nutrition in plants, and now pass on to notice shortly that of their Reproduction.

The granular matter, observed within the vesicles of the lowest Fungi, consists, as we have already mentioned, of germinal atoms. These gradually become of a larger size, and more distinctly vesicular; and then either they are discharged, by the bursting of the parent-cell, to commence an independent existence; or they germinate, in the form of buds, from its outer surface. Each separate cell or vesicle is capable of reproducing others like itself. Such is the case with the very simplest vegetable beings; but, as we rise in the scale of organic development, we find that some only of the cells are fertile or reproductive, while others serve solely for the nutrition of the plant. This arrangement exists in the numerous tribe of the Algæ, including the Confervæ. In certain species, the granules are either collected into groupes on the general surface of the *frond,* or on little separate leaflets, or inclosed in distinct cells, which may be regarded as analogous to the *Spores* of the higher groupes of the Cryptogamic class. In the Fucus *Vesiculosus,* or common bladder-wrack, the reproductive cells are evolved from a special receptacle, and separate altogether from the parent plant, when they are mature. It is in some of the Confervoid family that the curious phenomenon of Spontaneous Motion of the germinal granules is most conspicuous. At first, these atoms are observed as green dots adhering to the inner surface of the fertile cells; they gradually become more and more distinct, and acquire a more definite appearance, and then separate themselves from their point of attachment, and move about freely in the fluid of the cell. When they are discharged, by the bursting of the cell, they are single, and more or less globular in shape; but, by the evolution of new vesicles on their exterior, and the gradual elongation of these as they are successively formed, they begin to assume the filamentous configuration, characteristic of a Conferva.

In the proper Mushrooms, the fertile cells—or *spores* as they are called —which inclose the germinal granules, are collected together only in one part of the plant—viz, the fructifying membrane of the *pileus* or cap. The rest of the plant serves solely for the purposes of general nutrition and respiration, and has nothing to do with the reproductive apparatus, except in as far as the nourishment is thereby conveyed to it. Thus we observe that, as we rise in the scale of vegetable organisation, the Reproductive organs—which at first constituted the entire substance and body of the individual—form a smaller and smaller proportion of the entire plant, and their function is more and more independent of that of Nutrition. It may be here worth while to allude for a moment to the extraordinary productiveness of certain of the fungi: the number of germs, liberated from a single plant, almost defies calculation.

"Of this," says Dr. Carpenter, "any one may convince himself by examining a puff-ball in a state of maturity. On this subject Fries states, 'The sporules are so infinite (in a single individual of *Reticularia maxima* I have counted above 10,000,000), so subtle (they are scarcely visible to the naked eye, and often resemble thin smoke), so light (raised, perhaps, by evaporation into the atmosphere), and are dispersed in so many ways, (by the attraction of the sun, by insects, wind, elasticity, &c.), that it is difficult to conceive a place from which they can be excluded.' According to this view, then, the germs of all kinds of Fungi are constantly floating in the atmosphere; and one species or another developes itself, according as the nature of the decomposing matter is respectively adapted to each." 71.

The *Bovista giganteum*, a large Fungus of the puff-ball tribe, has been known to spring up in the course of a single night, from a mere speck or point to the size of a large gourd, which has been estimated to contain 47,000,000,000 cellules.

In the higher Cryptogamous plants, the spores are contained in cases or receptacles, termed *thecæ*. But the mere circumstance of this additional envelope makes no change in the essential character and constitution of the organ. Each spore is a *cell* which includes numerous granular atoms; and these atoms, when mature, are observed to float about freely in the contained fluid. When the process of germination commences, several minute tubular prolongations shoot out from the walls of the cell; these also are found on examination to contain moving green granules, which unquestionably are the germs of future growths or formative cells. By the gradual and successive development of lateral rows of new cells, the expansion, known as the *frond* of the plant, is at length developed: this process may be well watched in the *Marchantia polymorpha*. From the lower surface of the frond, radicle fibres proceed; while, on its upper surface, numerous *stomata* or air-apertures are formed.

The germination of the spores of the Ferns takes place exactly in the same way as of the more subordinate groupes of the Cryptogamous family. Although the one tribe of plants becomes subsequently much more highly organised than the other, there is no difference in the mechanism of their Reproductive apparatus.

Let us now briefly enquire what are the analogous parts in Flowering or Phænogamous plants to the spores of the Cryptogamic.

If we examine the particles of the *Pollen* dust of a flowering plant, we shall find that they are vesicles or *cells*, containing a semifluid matter that seems to be composed, in a great measure, of minute granular atoms: these atoms are often seen to be in active spontaneous motion. Here, therefore, we obviously have the analogue formation of the Spores in the lower tribes. But it may be asked, what then is the difference in the process of Reproduction in the two great sectional families of plants? The difference seems to consist in this simple circumstance—that the *spores* in the one are sufficiently organised in themselves to maintain an independent existence, and are therefore capable of germinating when separated from the parent; whereas, the *pollen-granules* of the other require to become more fully developed, and are for this purpose received into a receptacle (the ovule), there to be nourished until they are mature. The changes, that are observed to take place in these vesicular granules, when they are shed on the moist surface of the Stigma, appear to be in every respect similar to those which we have described to occur in the Spores of the *Marchantia*, when they begin to germinate. A number of tubular filaments are protruded from every vesicle, and each of these little tubes are found to contain some of the granular atoms which had existed in the vesicle itself. These gradually work their way along the Style, until they reach the Ovarium or Germen of the plant, where they are received into the ovules (on the surface of each of which there is a minute aperture) which are lodged within. According to this view of the subject, each germinal granule may be regarded as the nucleus of an ovular cell. Within this cell it is nourished with the mucilaginous and feculine fluid that has been already prepared for its use, and there it begins to form the first cells of the future embryo plant. In this, its primary, stage of development, a close analogy exists between the organisation of the young seed in the flowering plant and that of the humblest fungi, as the Red Snow and Yeast plants. As the cells multiply and increase laterally as well as lengthwise, we then perceive a structural arrangement that is strictly the same, as that which we have described to be present in the proper Algæ. Other resemblances may be traced in the ulterior phases of its growth. The Cotyledons may be regarded as the analogue formations of the fronds of the Hepaticæ and young Ferns; and this analogy is still stronger as regards the latter plants, for they (the cotyledons) are, it is well known, of merely transitory existence, just as the fronds are in the case of the Ferns.

Up to this point of its development, the young phænogamous plant is entirely composed of Cellular or vesicular tissue; and it is not until the plumule has risen to some height, and the rudimentary leaves begin to make their appearance, that any distinct traces of Fibrous or Vascular tissue can be perceived.

From the review, however imperfect, which we have now taken of the structure and functions of different tribes of plants, it must be obvious, we hope, that the doctrine of Cell-Formation is the key, as it were, to unlock the hidden mysteries of Vegetable Anatomy and Physiology. A stream of light begins to play on the surface of the dark and hitherto inscrutable wonders of vital action. The processes of Nutrition and Reproduction are shewn to be only modifications of a similar, if not the same, agency; and the apparently dissimilar forms of the various families of plants, from the highest of the Vascular to the very lowest of the Cellular, are found to be closely allied to each other, not less in the characters and development of their structure than in the mechanism of their leading functions.

Before dismissing the line of argument derived from the productions of the Vegetable Kingdom, there is one topic to which we wish briefly to allude, as it has some bearing on a pathological question, to be afterwards noticed.

By some Botanists it has been alleged that many of the Fungoid growths, which form on the surface of living plants, are to be considered rather as Diseases of the vegetable tissue on which they appear, than as distinct and individual plants. The Blight, Mildew, Smut, &c. have been compared to the Exanthemata in the human subject; and, in accordance with this idea, they have been ascribed to some irregularity or disorder of the stomata or pores on the surface of the plant. There cannot be a doubt that these *Fungi*—if we are still to call them so—are readily communicable from one plant to another, just as the contagious Exanthemata are. How their germs are introduced into the tissue of the living plant, has not yet been satisfactorily decided;—whether it is that they are absorbed by the Roots and conveyed along with the sap to different parts of its structure; or whether they at once enter the Stomata on the surface of the leaves, and there become developed. The latter is the more probable idea; and it receives confirmation from the singular and well-ascertained fact, that certain Insects, as well as plants, are subject to the formation of such fungoid growths within their bodies—the germs having become, (most probably) introduced into the tracheæ or breathing apertures on the surface of the body of the animal. Of this nature is the disease, termed *Muscardine,* to which silk-worms are liable. "This disease," says Dr. *Carpenter,* "has been ascertained to be due to the growth of a minute fungus, nearly resembling the common *mould,* within their bodies. It is capable of being communicated to any individual from one already affected, by the introduction, beneath the skin of the former, of some particles of the diseased portion of the latter; and it then spreads in the fatty mass beneath the skin, occasioning a destruction of this tissue, which is very important as a reservoir of nutriment to the animal, when it is about to pass into a state of complete inactivity. The fungus spreads by the extension of its own minute stems and branches,—and also by the production of new germs, which are taken up by the circulating blood, and carried to distant parts of the body. The disease invariably occasions the death of the Silk-worm; but it seldom shews itself externally, until afterwards, when it rapidly shoots forth from beneath the skin." The analogy, that has been traced between this vegetable morbid growth and some of animal origin, will be pointed out in a subsequent part of this article.

II. We now proceed to the second great division of our subject;—viz. to point out the illustrations which the Cell-formation Doctrine receives from the organisation, and mode of development, of animal bodies.

As in the Vegetable, so in the Animal kingdom, the simplest form of living being is that of an independent *cell* or membranous bag, having no distinction of parts, absorbing by every part of its surface, and propagating by the formation of small cells or bags, like to itself, within the parent cavity. This definition applies at least to the common *Hydatid* or Acephalocyst; and, if we are to regard this Entozoon as possessing an independent existence, it would seem that the presence of a mouth, leading into a central sac or stomach, is really not essential to an animal being, as has been generally asserted.

Some physiologists, however, and we observe that Mr. *Owen* is one of the number, view the Hydatid rather as a morbidly enlarged organic cell, than as an independent being. Let us hear what he says of it:—

"The Acephalocyst consists of a sub-globular or oval vesicle filled with fluid. Sometimes suspended freely in the fluid of a cyst of the surrounding condensed cellular tissue; sometimes attached to such a cyst; developing smaller acephalocysts, which are discharged from the outer or the inner surface of the parent cyst. These acephalocysts vary from the size of a pea to that of a child's head. In the larger ones the wall of the cyst has a distinctly laminated texture. They are of a pearly whiteness, without fibrous structure, elastic, spurting out their fluid when punctured. Their tissue is composed chiefly of a substance closely analogous to albumen, but differing by its solubility in hydrochloric acid; and also of another peculiar substance analogous to mucus. The fluid of the acephalocysts contains, according to Lobstein, a small quantity of albumen with some salts, including muriate of soda, and a large proportion of gelatin.

"The tunic of the acephalocyst is usually studded with more or less numerous and minute globules of a clear substance analogous to the 'hyaline,' whose remarkable properties in reproductive cells, Dr. Barry has recently described, and from which the young acephalocysts are developed. No contractile property, save that of ordinary elasticity, has been observed in the coats of the acephalocyst; no other organisation than that which I have just described; no other function than that of assimilation of the surrounding fluid by the general surface, and the development of new cells from the nuclei of hyaline." 45.

After comparing it to the Protococcus and the other lowest forms of Cryptogamic plants, which consist of a simple transparent cyst, he adds:

"The knowledge that we now possess of the primitive embryonic forms of all animals and of all animal tissues, places us in the position to take a true view of the nature of the acephalocyst. It seems to me to be most truly designated as a 'gigantic organic cell,' not as a species of animal, even of the simplest kind." 45.

Many physiologists, however, still regard the Hydatid as a genuine Entozoon, and certainly with considerable shew of reason; for its powers of reproduction, by the formation of gemmæ or buds between its layers, seem to entitle it to such a classification.

But not to insist upon this example, what, we may ask, is the structure of some of the lowest Infusory animalculæ? *Ehrenberg*, indeed, has imagined that, within the minute globular sac that constitutes the body of the animal, there is an intestinal canal consisting of numerous bags or stomachs, into which the food is successively received; hence the appellation of *Polygastrica*, which he bestowed on these beings. But the truth of this conjecture seems to be very questionable; and many of the best microscopists in the present day are of opinion that there is really no canal within for the digestion of the food, but that this is simply received into the common cavity of the body, and floats about loosely within it.

(We need not at present do more than merely allude, *en passant*, to those most beautiful fringed fibres that surround the orifice or mouth of the animal, and which, under the name of vibratile *Cilia*, have of late years attracted so much notice in physiological researches. The use, which they serve, is obviously to cause currents in the surrounding water, and thus to draw the particles of food towards the opening of the mouth.) Here therefore we have an animal formation that is not a little analogous with the Red-Snow and Yeast Fungi. In both cases, the living being seems to consist of nothing but a *cell* or vesicle,—in the one class indeed, and not in the other, this has an orifice or mouth for the reception of the food—which serves for the purposes alike of self-Nutrition and of Reproduction.

This therefore may be regarded as the primordial and elementary form of zoological development. The animal is a single cell, and nothing else. The first advance from this most simple state is when several such Cells are aggregated or conglomerated together, and so arranged as to form a continuous whole; but yet are so independent of each other that they can live apart, and form separate existences, when severed from the parent mass.

This is the case with the *Hydra*, or common fresh-water polypi, which exhibits the appearance of a soft membranous bag, provided with a mouth and a circle of arms or tentacula around it. These serve for the seizure of its food. This is drawn to the mouth by the contraction of the tentacula, and the currents in the surrounding water produced by the constant motions of the vibratile *Cilia*, with which they are fringed ; and thence it passes into the general cavity of the body, there to remain until it is digested, and afterwards to be rejected by the same orifice by which it entered. Every part of the inward sac or stomach seems capable of actively absorbing the nutritious fluid. This is then distributed in all directions, through the substance of the body—not indeed by any distinct vessels, but by mere passages or canals between the different cells that compose its substance.

Every one knows that a Hydra may be divided into numerous pieces—10, 20, or even more—and each of these pieces will become a separate and entire animal. This fact alone proves the independent vitality of the *cells*, which go to form it. But there is another circumstance, connected with the organization of the Polypes, that deserves our special notice in the present inquiry. There appears to be little or no difference in the active powers of the Inner and Outer surfaces of its body ; for it is well known that this little animal may actually be turned inside out like the finger of a glove, and yet all the functions of life will go on as well as ever. The Skin will act as the stomach, and the Stomach will do the duties of the skin.

How emphatically does this curious fact demonstrate the primordial identity of the Cutaneous and Mucous structures! As we advance in our inquiry, we shall meet with some interesting proofs of the close analogy that may be traced in this respect, even in the case of the higher animals. But this remark only *en passant:* let us proceed in our *ascending* examination of animal development.

As the *Hydra* consists of an assemblage of numerous Cells, which are more or less independent of each other, so the *Sertularia* and many other members of the Polypiferous family may be said to consist of an assemblage of Hydræ associated together into one, and yet each of them capable of living by itself. There is a stem and branches of a horny texture; and on the sides or ends of these are a number of little cells or bell-shaped chambers with their mouths directed upwards, in every one of which there resides a separate Hydra-like polype. Each of these Polypes is capable of living apart; but all are connected together by a set of channels or rudimentary vessels that pass along the stem and branches.

The next step of advance in animal organization is that exhibited in the structure of the *Sea Anemone* and the proper coral-forming Polypes. Their anatomy may be described thus:

The mouth opens into a rounded stomach, which has no second orifice; and, around this central sac, there is a series of radiating membranous partitions, which divide the space intervening between it and the outer covering of the body into numerous *cells* or chambers: these are destined to form and prepare the germs or ova of the offspring.

The structure of the *Acalephæ* or sea-nettles is very much the same. In the *Medusa*, for example, the semi-globular umbrella-shaped disk above contains the stomach, which is placed in the centre of the gelatinous mass and which opens by a single orifice or mouth, directed downwards. Around the stomach are four chambers, in which the eggs are prepared. In the edge of the disk, the nutritious fluid, which flows in channels excavated out of the soft tissues, seems to be aerated by being thus exposed freely to the surrounding water.

It is not until we come to the *Echinodermata* that we meet with any distinct traces of vessels or a circulating apparatus; and, even in them, this is still very imperfect. We may therefore regard the Echinodermata (which contain the highest members of the Radiate class) as forming the commencement of the *vascular* series of animals, and as being the analogues of the Mosses and Ferns in the vegetable world, in opposition to all the inferior zoological tribes whose tissues bear a very close resemblance to those of *cellular* plants, the fluid and solid parts being in contact with each other in every part of the body.

III. Having reached this point in our enquiry, we shall now direct our readers' attention to some of the most interesting facts, derived from the Embryological Anatomy of the higher animals, which serve to shew how intimately allied in structural organization they are, at an early period of their development, to the groupes we have been just considering.

The Ovum of all vertebrate, and of many invertebrate, animals may be described to be a compound *cell*, or a cell containing another cell within it. The outer one is called the Yolk-bag, or membrana Vitelli, and contains the yolk; the inner one is the Germinal vesicle, within which are numerous granular particles, that are the germs of new or embryo cells. The germinal vesicle seems to be the essential part of the ovum; the vitellus or yolk serving merely for the supply of nourishment to it, after impregnation. In many respects, it (the vesicle) may be regarded as analogous to the cell which constitutes the entire structure of the lowest Infusory animals. Mr. *Owen* quotes some interesting remarks of Dr.

*Martin Barry* on this very curious subject, and we cannot do better than present them to the reader. It may be useful to premise that the animalcule alluded to in the following obervations—the Volvox Globator—is one of the polygastric Infusoria. It is described as "having the form of a hollow globe or rounded cell with a transparent wall, including other smaller but similar globes. This wall is studded all over with minute green points, disposed at regular distances from each other; and, when these are examined with a good microscope, they are found to be separate animalcules, closely resembling in structure those which in other species are separate; each having its own *Cilia*, by the vibrations of which, in connexion with the rest, the whole mass receives a rolling motion. The interior globes are young structures of a similar kind, which have had their origin in one of the animalcules of the parent animal. This, by dividing and subdividing, forms a new groupe of animalcules, which is at first attached to the interior of the wall of the parent structure; but after a time it is separated, and floats in its cavity; and, when it is mature, it is liberated by the bursting of the wall that incloses it—just as is the case with the cells of the Red Snow Fungus;—the destruction of the parent being thus necessary for the propagation of the race. Not unfrequently a third generation may be seen within the second, previously to the setting free of the latter; and, under the brilliant light and high magnifying power of the solar microscope, even a fourth generation has been seen within the third."

We are now prepared to appreciate Dr. *Barry's* observations, to which we alluded.

"Between the appearance presented by the mammiferous germ during the passage of the ovum through the Fallopian tube, and those met with in the young Volvox globator while within the parent, I find a resemblance which is very remarkable indeed, extending even to minute details. Not only do the cells of which the young Volvox is composed form a body resembling a mulberry, with a pellucid centre, but the cells gradually increase in number, apparently by doubling, at the same time diminishing in size, like the cells of the mammiferous germ; which they resemble also in being originally elliptical and flat.

"Some of the points of resemblance now mentioned were recognised in the delineations of the Volvox given by Professor Ehrenberg; others were noticed during some observations I have myself made on this very interesting microscopic object. Professor Ehrenberg has figured five pellucid globules in a young Volvox just escaped from the parent. These, the germs of another set, evidently resulted from division of the pellucid mass visible in an earlier state: so that here is to be recognised fissiparous generation of the kind I have described as reproducing cells."—*Owen, p.* 24.

"Fecundation of the ovum takes place in the same manner as nutrition of the cell, and seems, in some instances at least, comparable to the nutrition of one of the Infusoria.

"But farther, I recognise in Ehrenberg's delineations of the Infusoria, not merely a cell-formation, but everywhere the existence of transitory or assimilative cells.

"And farther still: the infusorial cells, like the cells of the larger organisms, have their origin in globules which become discs or 'cytoblasts;' these passing through stages such as those of ordinary cells. Thus in Ehrenberg's *Monadina* are to be found, I think, the following grades, perfectly analogous to the grades of cells:—

"1. Globules and discs.

"2. Discs with a pellucid point.
"3. The point dividing.
"4. Nucleated cells.
"5. The nuclei dividing and thus giving origin to
"6. Young cells, which are seen both within and escaped from parent cells.

"There really seems to have been much truth in the remark long since made by Oken, that animals are groups of bodies comparable to the Infusoria. The cell is itself a little organism; and cells coalesce to form a larger one.

"The remarks just made respecting fissiparous generation, I apprehend, may be applied to gemmiparous reproduction, or propagation by means of buds."—*Owen, p.* 25.

Mr. *Owen* takes exception to the comparison alluded to in the concluding paragraph; for—while he admits that the minute Infusoria, which seem to have their development arrested at the first or nearest stage from the primitive cell-formation, offer close and striking analogies to the primitive cells out of which the higher animals and all their tissues are developed—he emphatically remarks that the very step, which the Infusory Animalcules take beyond the primitive cell-stage, invests them with a specific character as independent and distinct in its nature, as that of the highest and most complicated organisms. "No mere organic cell," he continues, "destined for ulterior changes in a living organisation, has a mouth armed with teeth or provided with long tentacula; I will not lay stress on the alimentary canal and appended stomachs, which many still regard as *sub judice.*"

The whole subject of Embryological Development is replete with remarkable illustrations of the law of Cell-formation. It would lead us greatly to exceed the limits of the present article, if we were to enter into a minute description of the successive stages or phases of this wonderful process; and this we regret the less, as we shall probably devote an article in our next number to its elucidation. A few explanatory remarks are all that we can find room for, just now.

The Yolk—and this, it should be remembered, is a mere magazine of nourishment stored up for the future embryo, just as the starchy and oily matter in the Seed is intended to serve for the germ of the plant—was discovered by *Schwann* to consist of delicate *cells* loosely aggregated, of oil globules, and of an albuminous fluid in which these are imbedded. He indeed is of opinion that it (the yolk) should be regarded not as mere nutritive matter, but rather as a body endowed with life; since the cells, of which it is made up, take an essential part in the formation of the embryo; but in this view he is not followed by most physiologists. That the cells effect a chemical change upon their contents during the process of Development, is obvious from the fact that the yolk then loses its coagulable property. *Reichert* has discovered that the formation of young cells, within the previously-existing cells composing the entire mass of the yolk, goes on continually during the whole period of development in frogs, in which tribe of animals the entire yolk is employed in the building-up of the embryo.

According also to the interesting researches of Dr. *Barry* on the changes observable in the ovum of the rabbit, just before and immediately after Impregnation, there is a constant formation of *cells* going on in the centre of the yolk. Each layer of these cells, while they enlarge and assume a flattened elliptic shape, is pushed outwards by a new set that are developed internally to them. After impregnation, when the germinal vesicle has passed to the centre of the ovum, the development of cells, here described, takes place in successive layers around that body. Every layer, on reaching the exterior of the yolk mass, is seen to be circumscribed by a proper membrane, and at length undergoes liquefaction; the layer within, or internal to it, then supplying its place. Each of the flattened elliptic cells also contains minute cells, arranged concentrically around a pellucid point; and the contained cells again present a similar arrangement. The same process, in fact, which is observed to occur in the yolk as a whole, seems to take place in all the cells, of which it is composed.

While these changes are going on in the substance of the Yolk, the germinal Vesicle also—the essential part of the Ovum—is the seat of no less active evolutionary movements. Its centre is invariably found to exhibit (at a certain period) a dark nucleus. This gradually becomes larger and larger, and at length it is seen to contain a cavity filled with a most pellucid fluid. The outer portion of the spot or *nucleus* resolves itself into cells; and the foundations of other cells come into view in its interior, arranged in layers around the central cavity; the outer layers being pushed forth by the continual origin of new cells in the interior. The latter commence as dark globules in the pellucid fluid. The central portion of the altered spot, with it pellucid cavity, was at first situated towards the surface of the germinal vesicle; but, after impregnation, it passes to the centre of it, and the vesicle itself returns to the centre of the ovum. It is believed that some vivifying substance, probably a Spermatozoon, has by this time entered into the cavity of the germinal vesicle from the exterior of the ovary—as a fissure has sometimes been seen in the membranous envelope of the ovum. Dr. *Barry* proceeds to describe how two cells, larger than the rest, make their appearance at the centre of the altered germinal spot: these two cells are the earliest rudiments of the future being. They enlarge, and absorb the fluid of the surrounding cells, which, as well as the germinal vesicle itself, gradually melt away and cease to exist. From them, as from a centre, other cells are evolved, till the germ acquires a mulberry-like appearance, and the number of the cells has increased so much that they cannot be counted. Within each, there is an intussusception or formation of new cells always going on; and thus there is a continual repetition of the very same phenomena which were first observed in the primordial Cell or germinal Vesicle. The embryotic mulberry-like mass subsequently passes from the centre of the yolk to a certain part of the vitelline membrane, which by this time has become invested with a layer of cells:—by the coalescence of the two the future amnion seems to be produced.

Fortunately it is not at all necessary for our present purpose to pursue this intricate subject further, as it is scarcely possible to render it at all intelligible without the aid of pictorial illustrations. There is one circumstance that deserves especial notice before we conclude, as shewing the independent vitality of the Embryo, even after it is considerably developed:—the Ovum, while it is undergoing its earliest phases of formation within the uterus, lies quite free and unattached in the cavity of this organ. Subsequently, indeed, the internal surface of the womb becomes covered with an exsudation, which is at first composed of numerous cellules, and constitutes the Membrana Decidua. Into this soft spongy tissue the *villi*, growing from the chorion of the ovum, become inserted, and seem to act as rootlets, drawing nourishment from the maternal structure, without having any organic vascular connection with it. At a later period of pregnancy, the union between mother and child is effected by means of the Placenta; which, if it does not establish a direct vascular communication between them, at least serves to render the latter much more dependent on the former, than hitherto it had been. Before dismissing the embryological argument of our question, we gladly avail ourselves of one or two extracts from Mr. *Owen's* lectures, which will be read with interest by every one. Treating of fissiparous Generation, he remarks;

"The Monad divides itself before our eyes, constituting two, then four, next eight individuals, and so on. The impregnated germinal vesicle, which the Monad permanently represents in nature, propagates, in like manner, by spontaneous fission and assimilation, a number of impregnated cells like itself. Most of these cells are metamorphosed into the tissues of the growing embryo, but not necessarily all. Certain nucleated cells, the progeny of the primordial one, and inheriting its powers, may become, without further stimulus, the centres of development of processes like those which have built up the body that contains them: they may bud forth from the stem of the Hydra, and form new individuals by the process of gemmation; they may bud forth, in like manner, from the larval polype of the Medusa, which thereby procreates in its immature, and, as it seems, virgin state, like the wingless larvæ of the summer Aphides; they may enter the unimpregnated oviducts of these insects, and be there developed in the manner which has already been described." 367.

And, after shewing that *Geoffroy St. Hilaire* and some of his disciples have pushed too far their favourite dogma that the embryos of the higher animals pass through, during the course of their development, a succession of phases, each of which is exactly represented by the permanent and mature organization of the lower tribes, he says:

"The extent to which the resemblance, expressed by the term 'Unity of Organisation,' may be traced between the higher and lower organised animals, bears an inverse ratio to their approximation to maturity.

"All animals resemble each other at the earliest period of their development, which commences with the manifestation of the assimilative and fissiparous properties of the polygastric animalcule: the potential germ of the Mammal can be compared, in form and vital actions with the Monad alone, and, at this period, unity of organisation may be predicated of the two extremes of the Animal Kingdom. The germ of the Polype pushes the resemblance farther, and acquires the locomotive organs of the Monad,—the superficial vibratile cilia,—before it takes on its special radiated type. The Acalephe passes through both the Infusorial and Polype stages, and propagates by gemmation, as well as spontaneous fission, before it acquires its mature form and sexual organs. The fulness of the unity of organisation which prevails through the Polypes and larval Acalephes, is diminished as the latter acquire maturity and assume their special form." 368.

There is a very interesting question of inquiry, connected with the early development of the embryo, and one that bears very directly on the physiological doctrine, which we have been endeavouring to explain—we mean; How, and at what time, is the blood formed in the nascent being?

It might naturally be supposed that the Vessels, which are to contain the fluid, should be formed first. But this is not the case: the blood is generated, before the slightest traces of vascular tissue can be discerned. The sole material for its formation is the vesicular substance of the Germinal Membrane; and this itself is developed at the expense of the yolk of the ovum.* Around the central transparent part of this membrane, where the rudimentary embryo is observed to be, there is an opaque ring, which has been called the 'Area Vasculosa,'—because within it the blood and blood-vessels are formed. The earliest trace of the former presents the appearance of a mere granular deposit; this gradually becomes arranged in insulated portions or islets, having transparent interspaces between them. In these interspaces a fluid, that is at first yellowish, and is afterwards of a red colour, is observed: this is the Blood.

According to the observations of *Schwann*, the Blood-Vessels are developed from nucleated cells, which send out processes from their sides; these processes from the different cells gradually unite together, and in this way ramifications and a network are produced. Vessels then begin to extend from this network in the 'Area Vasculosa' into the transparent central spot, and join the rudimentary Heart; which at this time is nothing more than an elongated tube. When the heart begins to pulsate, the fluid, which circulates, is at first nearly colourless: the red globules or corpuscles only appearing subsequently. Physiologists are not agreed as to whether the blood, when first formed, has any power of self-propulsion, and whether it moves in the 'Area Vasculosa' of the germinal membrane, before the rudimentary heart has begun to act. Numerous arguments, both *pro* and *con*, have been urged with great ability by very distinguished writers. The movements of the Sap in plants have often been appealed to, in favour of the idea that the Blood has an inherent power of self-propulsion. But, as *Müller* remarks, "although the circulation of the vegetable sap, effected by means of attraction only, shews the possibility of the occurrence of a similar phenomenon in animals, still there are at present no direct observations which prove it in a conclusive manner. The circular currents in many of the lower animals, which had been compared to the motion of the sap in the Chara, and the movement of fluids in vessels, themselves destitute of motion, observed in some Distomata by *Ehrenberg*, have been recently shewn to depend on the vibrations of cilia." The question may therefore be regarded as still "sub judice."

This seems to be a fit opportunity to introduce a few remarks on the Globules of the blood, in connection with the doctrine of Cell-formation. The blood of all Vertebrate or red-blooded animals consists of two

---

* The following important remarks by *Müller* well deserve especial attention. "It is evident," says he, "that, in the embryo, the Blood is formed from the substance of the Germinal Membrane, which assimilates to itself the fluids of the ovum, and that no particular organ is then required; for at that period no organs, such as intestinal canal, liver, spleen or lungs, exist. This fact teaches us that we must not expect to discover the process of the formation of the blood and its red particles (from the globules of the chyle?) in any special organs of the adult animal; indeed it is very probable that, in the adult, the chyle is converted into blood under the influence of the same general vital properties, which are in action in the incubated egg."

distinct parts, viz: a clear and nearly colourless fluid,—known by the name of the *Liquor sanguinis* or *Plasma*—and an innumerable number of rounded particles floating in this fluid, well known as the Globules of the blood. The former is a solution (?) of the fibrine or coagulable lymph in the Serum or watery portion; while it is to the latter that the colour of the blood is altogether owing. These globules are always most abundant in animals of great muscular vigour and activity, and which consume the largest amount of oxygen during respiration: hence, they are most numerous in the blood of Birds, and least so in Reptiles and Fishes. In the human subject, their proportion varies very much according to the health of the individual; abounding in robust constitutions and being much diminished in pale and feeble habits. When examined with the microscope, they are found to be flattened *cells* or vesicles, which contain a red-coloured fluid, and a *nucleus* or assemblage of minute granules, adherent to each other and to the parietes of the enveloping saccula.

This nucleus " corresponds in all essential particulars to the nucleus of the Vegetable Cell, as well as to that of the cells, in which the animal tissues have their origin." The contained fluid of the red globules is found to have a portion of *iron* dissolved in it; and, from a variety of considerations, it has been very reasonably conjectured that the presence of the ferruginous salt is for the purpose of absorbing oxygen—either from the atmosphere or from water—during the process of respiration.* Hence the red globules have been supposed to serve the purpose of carrying or conveying Oxygen from the lungs to all the various tissues of the body; and of bringing back the Carbonic acid (in the form of carbonate of iron), that is continually generating in the capillary vessels, to the lungs, to be there disengaged during expiration. As there are no red globules in the blood of the Invertebrata (though not a few possess great muscular activity), and as these animals nevertheless require the introduction of oxygen into the system quite as much as those of the higher zoological class, there must be some means provided to compensate for the deficiency in question. It has been supposed that the numerous *tracheæ* or air-tubes, which permeate every part of the body in Insects and others of the Articulate family, may serve for this purpose.

Mr. *Owen* was the first, we believe, to remark that the red Globules of the blood, like other organic Cells, and like the minute Infusory animalcules, may be multiplied by what is called fissiparous generation; in other words, by their spontaneous division into separate portions, each of which ultimately forms a distinct and independent globule. The notion of *Eber* and *Mayer*, that the red particles are really Infusory animals, is entirely fanciful. There can be no doubt, however, that they are continually dying and reproducing themselves, in a way very similar to what takes place in the simplest forms of animal existence.

Besides the red globules now described in the blood of Vertebrate Ani-

---

* We need scarcely remind our readers how important a remedy *iron* is in all cases where there is a deficiency of the red globules. Being an essential ingredient in the fluid which they contain, it seems to have the power of accelerating their reproduction in cases where they are deficient, as in Chlorosis, Anæmia, &c.

mals, there are others which are nearly destitute of colour, and which have been supposed to perform a very different function. "Their size," we borrow the description from Dr. Carpenter's Cyclopædia, "is pretty much the same in all the Vertebrata, (that of the proper red globules varies exceedingly, being much larger in Reptiles and Fishes than in the Mammalia), and is usually about 1-3000th of an inch in diameter. In the blood of man and the mammalia in general, they are not easily distinguished from the red particles; since their size is so nearly the same; and the colour of single disks of the two kinds is not very dissimilar. But, with a good microscope, several differences can be seen; and there is much reason to believe that these colourless particles are the same with the particles of the chyle and lymph. In the lower Vertebrata, whose blood has large oval red particles, the difference between the two kinds is very obvious; and the resemblance, which the colourless globules bear to those of the chyle and lymph, is very striking, Similar colourless particles exist, to a variable amount, in the nutritive fluid of Invertebrated Animals; so that in this, as in some other respects, that fluid bears a stronger resemblance to the chyle and lymph of the Vertebrata, than it does to their blood, which is characterized by the presence of the red particles."

As we shall afterwards revert to the constitution of the Chyle and Lymph, we proceed now to notice briefly the manner in which some of the most important tissues of animal bodies are supposed to be formed from the elements of the blood.

When the Fibrine, that is contained in the *liquor Sanguinis*, is poured out upon any living surface, it forms a membraniform layer—which is known by the name of Coagulable lymph, in cases of inflammation and recent wounds. In this simple manner, it is imagined that the various *fibrous tissues* of an animal body have been originally formed. Of these, the most simple and the most abundant is that which has usually been called the Cellular Tissue, but which might be much more correctly denominated Areolar or Reticular Tissue,—to prevent confusion with the use of the words *cell* and *cellular* in the acceptation of vesicular, or consisting of vesicles or globules.

It is composed of a net-work of fibres and membranous laminæ, so conjoined and laced together, as to leave numerous interstices or intervening cavities, which are not closed, but communicate pretty freely with each other. By means of this tissue, the various structures and organs of the body are united together; besides, it enters more or less largely into the composition of every one of them.* The Serous membranes appear to be merely a modification of the Areolar tissue; and the same may be said of the Ligaments, Tendons, and Fasciæ—all of them consisting of mere fibres, which are more or less closely adherent.

---

* An interesting circumstance, connected with the formation of Cellular Tissue is that, when other parts of the body become wasted from loss or suspension of their functions, their organisation usually *degenerates*, and assumes its character. This is often seen to be the case, when muscles have been long disused, and when nerves have become paralytic. A similar change occurs in blood-vessels that become obstructed, and in glands, such as the Thymus, that waste away.

Besides these parts, it would seem that the outer layer of the true Skin and the proper substance—the woof and web, so to speak—of all Mucous membranes, as well as the internal coat of the Blood-vessels, consist of thin layers of membrane, composed of consolidated fibrine. This, of late years, has been called the *basement* or *primary membrane;* it is chiefly remarkable for the facility with which fluids can permeate it, although no pores are visible on its surface. In the case of the skin and mucous tissues, it is covered with an investing layer, known as the Epidermis and the Epithelium, whose structure we find, on microscopic examination, to be essentially different from that of the membrane on which it rests.

The *Epidermis,* when examined under a tolerable magnifying power, is observed to consist of numerous layers of minute scales or laminæ, which can be readily separated by brisk friction with any moderately rough substance.*

These scales are produced by the desiccation of the innumerable vesicles or *cells,* of which the Epidermis primarily consists. The inner layers of the cells, being kept soft by the moisture which transudes through the true skin, retain more or less distinctly their vesicular character; and, as the outer ones dry and fall off, these are pushed forward to supply their place. There is thus a constant decay and renewal of these Epidermal Cells, the strata of which are in various stages of development, in proportion to their proximity to the external surface. *Henle,* to whom we are indebted for the most accurate information on this subject, says, that the deepest stratum or layer consists of nucleated cells, in which the external vesicle is so little larger than the nucleus, that often it cannot be detected, and is sometimes, perhaps, really absent. Further from the surface of the Cutis vera, or, more externally, the nuclei appear more granular and of a larger size, while the cells themselves have enlarged in a still greater degree. Towards the outer surface, both nuclei and cells are so flattened as at length to have the form of delicate laminæ; and in the most superficial laminæ, the nuclei are scarcely visible. It hence appears that the nuclei of the *Epidermis Cells* are first formed; that the cells or vesicles are afterwards developed; and that they subsequently undergo certain changes of form, by which they are converted into the lamellæ, which we have described above. The part, that is called *Rete mucosum,* has usually been regarded as a distinct tissue or membrane. This is not correct; as, in truth, it is nothing more than one stratum (the last formed) of the epidermal cells, many of which contain a Pigment-secretion, to which the hue of the skin is owing. All the various modifications, which the Epidermis undergoes in different animals, exhibit, at some period of their development, more or less distinctly their primordial vesicular structure. Thus, in many shells, the external layers are observed to be formed of cells, arranged side by side, and filled with mineral matter; this is the case with the brownish substance, which constitutes nearly the entire shell of the Pinna, the edges of the outer layers of the common Oyster, &c. The

* Any one, who has been in the habit of using the horse-hair gloves, now so much in vogue, must have observed what a quantity of scurfy dust, or small scales, may be beaten out of them, after the body and limbs have been well curried.

scales of fishes and reptiles, the feathers of birds, the hoofs, claws, nails and hair of mammal animals, are all to be regarded as mere modifications of Epidermal tissue, and consequently exhibit the same organisation, when they are first developed.

The *Epithelium*, that invests the free surface of all mucous membranes, is essentially the same in structure as the Epidermis. Its surface is always found to be covered with a layer of minute cells—the *Epithelium-Cells*, that we believed to play so important a part in the process of Secretion. As in the case of the Epidermis, these cells are in a state of being constantly renewed; the outer layer bursting and discharging their contents, and an inner one coming forward to supply its place. This constant renewal is owing to the development of new cells, from germs contained in the *basement membrane*, at the expense of the "liquor sanguinis," which transudes through it from the vessels beneath.* The Epithelium-cells are believed to be the agents, by which the secretion of the *mucus* is performed. The mucous membrane, whose use is limited to this purpose, and which has no absorbing function at the same time,—such as that which lines all the respiratory passages,—is of a much more simple structure than that which performs both duties,—as, for example, the lining of the alimentary tube. In the case of the latter, the expansion of the mucous surface is prodigiously increased, not only by means of numerous folds and projections, but also by the pits or follicular depressions, dispersed along its whole extent. The function of *Absorption* appears to be performed by the Epithelium-Cells of the Villi which cover the former; while that of *Secretion* is performed by those Cells which line the latter.

---

* The following excellent description, by Dr. *Sharpey*, of the mode in which the blood serves for the nourishment and development of cells, will well repay an attentive perusal:—

"In the instance of cuticle and epithelium, no vessels enter the tissue, but the nutrient fluid which the vessels afford penetrates a certain way into the growing mass, and the cells continue to assimilate this fluid, and pass through their changes at a distance from, and independently of, the blood-vessels. Whether, in such cases, the whole of the residuary blastema remains as intercellular substance, or whether a part is again absorbed into the vessels, is not known. In other non-vascular tissues, such as articular cartilage, the nutrient fluid is doubtless, in like manner, conveyed by imbibition through their mass, where it is then attracted and assimilated. The mode of nutrition of these and other non-vascular masses of tissue may be compared, indeed, to that which takes place throughout the entire organism in cellular plants, as well as in polypes, and some other simple kinds of animals, in which no vessels have been detected. But even in the vascular tissues the case is not asbolutely different; in these, it is true, the vessels traverse the tissue, but they do not penetrate into its structural elements. Thus the capillary vessels of muscle pass between and around its fibres, but they do not enter them; still less do they penetrate the fibrillæ within the fibre: these, indeed, are much smaller than the finest vessel. The nutrient fluid, on exuding from the vessels, has here, therefore, as well as in the non-vascular tissues, to permeate the adjoining mass by transudation, in order to reach these elements, and yield new substance at every point where renovation is going on. The vessels of a tissue have, indeed, been not unaptly compared to the artificial channels of irrigation which distribute water over a field; just as

The Villi have been aptly compared to the *Spongioles* of the roots of plants, and seem to be endowed with the peculiar property of selecting the nutritive fluid and conveying it to the lacteal absorbents. These vessels do not reach to the extremity of the villi, nor do they open on the surface of the mucous membrane by any appreciable apertures; but the end of each villus is composed of a loose spongy tissue, in which a number of *cells* may be seen, in various stages of development, during the process of absorbing the chyle. It appears almost certain, from the recent observations of Mr. *Goodsir,* that the Absorption of chyle is really performed by these cells; and that a fresh crop (as it were) is being produced, every time that digestion takes place and chyle is prepared. In the interval, no cells can be seen in the ends of the lacteals; but they begin to be developed (from the germs that were left behind by the previous crop,) as soon as the chyle is prepared. Their growth is very rapid and their life is transitory. In growing, they absorb into themselves part of the fluid that surrounds them; and it is probable that, when they are mature, they either burst or dissolve away, and deliver this fluid to the absorbent vessels.

As we have already spoken of the Blood, it will be convenient now to notice the physical characters of the Chyle, and, with it, of the Lymph. Both of these fluids contain *globules* or floating Cells, and also fibrine; but they are more abundant in the former than in the latter. The chyle contains a large quantity of fat in a free state; while in the lymph and also in the blood, it is in a state of combination with other matters. The chyle, like the blood, contains *iron,* which it derives from the food; but the metal appears to be in a state of much less intimate combination in the former than in the latter fluid; for its presence can be much more easily detected by chemical re-agents.

The appearance and characters of the Chyle differ, in some important respects, according to the part of the lacteal system, from which it is obtained for examination. The following passage, from Dr. *Carpenter's* popular work, presents a good picture of these changes.

"If obtained," says he, "near the surface of the intestines, before it has passed through the mesenteric glands, it is entirely destitute of that power of spontaneously coagulating which is so remarkable in blood; and, when examined with a microscope, it is seen to present a number of oily globules of various sizes, together with an immense number of very minute particles or molecules, which also seem of a fatty nature; and to these last, whose diameter is between $\frac{1}{24000}$th and $\frac{1}{36000}$th of an inch, the milky whiteness, which characterises chyle, seems principally due. But the chyle, drawn from the lacteals, after they have passed through the mesenteric glands, possesses the power of coagulating slightly: hence it is evident that some of the albumen has undergone a transformation

---

the water penetrates and pervades the soil which lies between the intersecting streamlets, and thus reaches the growing plants, so the nutrititious fluid, escaping through the coats of the blood-vessels, must permeate the intermediate mass of tissue which lies in the meshes of even the finest vascular network. The quantity of fluid supplied, and the distance it has to penetrate beyond the vessels, will vary according to the proportion which the latter bear to the mass requiring to be nourished."

into fibrine. At the same time, a great increase is observed in the number of certain floating *cells*, which are occasionally to be noticed in the first chyle, but which are very abundant in the fluid drawn from the glands, and from the lacteals that have passed through them; these are colourless, and, like most other cells, contain smaller particles within them: their average diameter is about $\frac{1}{4000}$th of an inch. By the time that the chyle reaches the central receptacle (the thoracic Duct) its power of coagulating has still further increased; so that its resemblance to blood, except in regard to colour, is much stronger. The proportion of Fibrine and Albumen, which it contains, is much greater than that which existed in the first chyle, whilst the amount of oily matter is less; hence it seems probable that the latter has been partly transformed into albumen."

We shall not, at present, inquire what is the most probable cause of these successive changes in the constitution of the Chyle. Suffice it to say, that the accuracy of the preceding remarks has been amply confirmed by the most careful observations, and that the important and essential phenomenon in these changes is the gradually increasing number of the *floating cells*, as the Chyle approaches the thoracic duct.

In the Invertebrata, no lacteals nor lymphatics are discoverable in any part; the blood-vessels have therefore to perform the function of absorption. We have already stated that the blood of these animals is destitute of red globules, and has more resemblance to the chyle than to the blood of the Vertebrata.

Having thus briefly sketched the most remarkable features of the process of Absorption, we shall now, for a moment, turn to that of Secretion. As stated above, there is good reason to believe that the mucus of the intestines is secreted by the minute cells, which line the mucous membrane and the follicles dispersed upon its surface. These *Epithelium*-cells are continually coming forward, bursting and discharging their contents, and are then replaced by a deeper-seated new crop, which advance to maturity, as the others pass away. The mucous *follicles* seem to serve the purpose of merely increasing the extent of the secreting surface, at those parts where a large amount of secretion is required.

But it is not the secretion of Mucus only that is effected in this way. All the other Secretions of the body appear to be performed in a similar manner, viz: by the agency of cells lining mucous surfaces; such surfaces being in the form either of tubes or of rounded follicles. All the conglomerate Glands are ultimately resolveable into an innumerable number of *acini* or bundles of follicles, which open into minute efferent ducts: by the union of these ducts the main excretory duct of the gland is formed. Such a gland, therefore, when freed from its blood-vessels and the cellular tissue which enters so largely into its composition, would resemble a bunch of grapes; each separate grape attached to its own little footstalk, and these gradually coalescing and forming the main stalk which upholds the bunch.

This description accurately represents the structure of the Liver in Crustaceous and Molluscous animals. In Insects, on the other hand, the hepatic organ consists of a few elongated *tubes*, and there is no vestige of any proper glandular or parenchymatous substance; while, in some of the Polypes, the only rudiments of a liver are a few mere *follicles*, lodged in the walls of the stomach, and which therefore are quite similar to the intestinal follicles in the Mammalia.* These follicles,—whether simple as in the Zoophyte, or complex and conglomerate as in the higher animals—are believed to be invariably lined with *Cells;* which, by their constant formation and destruction, perform the important office of eliminating the particular secretion. We cannot indeed tell, nor even form a reasonable conjecture, why one set of cells should secrete Bile, another Mucus, a third Urine, and so forth. All that we can say is, that such is the fact; and that certain cells are endowed with the vital power of selecting from the circulating juices the particular elements of the secretion, which they are appointed to accomplish. The *why* is a mystery; but not a greater one than to explain how one portion of the petal of a flower should secrete a red dye, while the adjoining portion may secrete a yellow; or how some of the cells in the early embryo become converted into muscular substance, and others into cartilage or epidermis. Although much remains to be discovered, still it is an important point gained in our investigation of the process of Secretion, if we have ascertained with tolerable certainty that it is performed by the agency and interposition of *cells*.

A curious circumstance connected with this very important function is that, in certain states of the system, a Secretion is sometimes eliminated, not by the usual apparatus destined for this purpose, but either by another gland or by another secreting surface. For example, the Urine has been known to pass off by the skin, or the bowels, when an obstruction has existed either to its secretion by the kidneys, or to its escape from the bladder; and the Milk has occasionally been discharged from the salivary glands, kidneys, &c. when something had occurred to prevent the mammæ from doing their duty.

This interchange, or vicarious performance, of function will probably surprise the reader less, when he calls to mind the intimate analogy that exists,—not only in many of the lower groups of animals, but also in the early stages of the developement of the human fœtus,—between the cutaneous and mucous surfaces, and the very close resemblance in the mechanism, or elaborating apparatus, of the different secretions of the body. We have seen that there is good reason to believe that this invariably consists in the formation of minute *cells* or vesicles upon an epithelial surface, and that every gland, however complex its structure may be in the higher tribes of animals, is resolvable into one or more simple follicles or tubes. This identity of structure, therefore, partly prepares us to anticipate that one gland may occasionally perform the duties of another. Until we know more of the Chemistry of life and the elective attractions of living organisms, we cannot expect to push our inquiries much further.

There still remain one or two tissues or component parts of an Animal body, to whose development and formation we wish to allude, as affording

---

* As another curious illustration of the fact, that form or outward structure of a gland has nothing to do with determining the nature of the secretion which it elaborates, we may mention that, while the urine is generally secreted in *tubes*—which are either closely conglomerated and associated together, as in the medullary substance of the kidney in the Mammalia; or are separated and detached from each other, as in Insects—yet in Molluscous animals, this secretion is effected by mere *follicles*.

additional illustrations of the great Physiological Doctrine we have been considering: and first of the *Fat* or Adipose substance. This is to be regarded as a simple secretion that has been eliminated from the blood, and which is stored up in numerous sacs or *cells*, for the use of the individual organism. These cells are independent and distinct; they do not communicate with each other; and their function seems to be to separate from the circulating fluid the oleaginous particles, which constitute the Fat. The deposition, therefore, of this substance is strictly and truly a Secretion; and indeed a marked analogy may be traced between this process in animals, and the elaboration of the resinous and oily matter in the leaves of many plants; not only as regards the chemical proportions of the substances in question, but also because, in both instances, they are stored up in the cells which formed them, and then constitute part of the general fabric of the individual—not for the purpose of subsequent Excretion, but rather for that of self-Sustentation.

The *pigment* also, or colouring matter of the skin and *choroid coat* of the Eye is found on examination to consist of numerous *cells*, which have the power of secreting a black granular matter in their interior.

The black-spots on the skin of the Frog, Water-newt, &c. are produced by the presence of similar pigment-cells; the colouring matter being secreted by and stored up in them, just as the fat is in the cells of the adipose tissue.*

If space permitted, we might now notice at some length the development and formative evolution of the Solid parts of the body; but this part of our subject we can only glance at. The structure of nascent *Cartilage* is always appealed to, as affording a good illustrative example of the Cellular Doctrine. With the aid of the microscope, it is readily perceived to be composed of numerous *cellules* lying near together in the midst of a quantity of an inter-cellular substance, that is very similar to what exists in the general tissue of many plants. The conversion of cartilage into *bone* is effected by the deposition within the cells of earthy matter, that has been eliminated from the blood.

The following observations on the subject, by *Müller*, may be aptly quoted here:

"The process of the development of Cartilage seems to be independent of blood-vessels, and to be wholly analogous to the process of growth in vegetable tissues. How the Canals, radiating from the corpuscles of ossified bone, are developed, is not known. Two hypotheses are proposed by *Schwann*. If the osseous corpuscles are the cavities of cells, the thickened

---

* "The secreting cells not unfrequently possess the power of elaborating a peculiar colouring matter, either separately or along with the substances which seem more characteristic of the secretion. Thus the *ink* of the Cuttle-fish is in reality its urine, charged with a quantity of black matter formed in the pigment-cells that line its ink-bag; aud the corresponding secretion in other Mollusca is rendered purple by the same cause. The bile seems to be universally tinged with a yellow colouring matter, which may be regarded therefore as an essential part of the secretion; in some herbivorous quadrupeds it has a green hue, and the colouring matter by which this is given, appears to be identical with that contained in the vegetable tissues on which they feed."—*Carpenter.*

walls of which have coalesced with each other and with the inter-cellular substance so as to form the mass of the cartilage of the bone, then the radiating canaliculi must be regarded as canals, extending from the cavities of the cells through their thickened walls, and would be analogous to the pore-like canals of some vegetable cells. But if the osseous corpuscles are the cells themselves, and not merely their cavities, (the whole substance between the corpuscles being intercellular tissue), in this case the canaliculi will probably be radiating prolongations of the cells extending into the inter-cellular substance. According to this latter view, which *Schwann* regards as the more probable, the canaliculi would correspond to the processes given off from some cells of plants."

The *Nervous* and *Muscular* tissues also are primarily evolved, in the early Embryo, as a mere agglomeration of *cells*. These cells by apposition and coalescence first form tubes; and then these tubes are subsequently filled with the substance peculiar to each, and become so very closely and intimately coherent that the tissues, when fully developed, exhibit no appearance of their original cellular structure. The ultimate reason for such a transformation is thus ingeniously propounded by the talented author of the Cyclopædia:

"It is easy to see that, so long as cells remain isolated from each other, they exist as so many distinct individuals—performing, it may be, the very same operations—but doing this independently of one another. Now the very nature of the *Animal* (contrasted with the *Organic—Rev.*) functions requires that the actions of the several parts of the tissues, which perform them, should be most intimately connected; thus, when an impression is made upon any part of the surface of the body, it has to be *instantaneously* communicated to the *Brain*; or an effort of the will, acting through the brain, has to call into *immediate* operation a large amount of muscular tissue. We could not conceive these functions to be performed by a number of isolated *cells*; and we find in fact that the muscular and nervous tissues are composed of *tubes*, containing substances that are peculiar to each respectively. These tubes originate like the *ducts* of plants, in cells laid together end to end, the partitions between which have broken down; and the deposits that are found within them, on which their peculiar properties seem to depend, are formed at a subsequent time."

IV. In the last division of our subject, we intended to point out the several applications that have been made of the Cell-doctrine to pathological inquiries, and to the illustration of various diseases. Neither space nor supply of materials permit us to enlarge at present upon this most interesting topic. All that we can do is simply to notice those morbid states and changes of the body which (it has been supposed) are produced by the presence of extraneous, so to speak, and abnormal *cells*, either in a part or throughout the system generally.

From the researches of that distinguished physiologist, Professor *Müller*, it appears that such growths as *Cancer*, *Fungus Hæmatodes*, *Sarcoma*, *&c.* are essentially cellular or vesicular in their structure; the morbid *cells* being produced by a transformation of the natural and normal cells of the part into others of a new and deleterious character. These morbid cells seem to be endowed with the same power of self-Reproduction, which we have shewn to belong to the usual formative cells of all organised bodies; and indeed the very character of *malignancy* of the diseases in question is, in all probability, owing to the tendency which such cells have to multiply, and to make their appearance in another part of the body, if removed from their original *foyer*. This pathological doctrine cannot be considered as altogether novel; for it is only a modification—although unquestionably an important one—of the opinion of Drs. *Adam* and *Baron*, who attributed the formation of *cancer* to the presence of an hydatidiform body, which they called *hydatis carcinomatosa*, and of that of Mr. *Carmichael*, who supposes that it is owing to the develcpment of a body possessing an independent existence in those parts of the frame, whose vitality has become enfeebled, and the organised matter of which begins to be decomposed. The following observations on this subject by Dr. *Carpenter* well deserve an attentive perusal.

"The presence of cells or vesicles containing nuclei, the walls of which cells are easily separable from one another, is an almost constant characteristic of these growths; and thus we may regard them as not having advanced far in the process of organization. These cells frequently become elongated, so as to present the spindle-form; and, when such cells are regularly arranged in clusters, the tissue will possess more or less of a fibrous character. In no instance has the complete transformation of the cell itself into a bundle of fibres been observed; so that the whole tissue may be regarded as developed upon the low and simple type of the vesicular tissue of the lower plants and animals. Each cell enjoys, as it were, a separate individuality, so long as it is supplied with nourishment prepared for its assimilation; and effects the multiplication of its kind by the development of new cells of its own nature within itself." After alluding to the analogy that may be traced between the formation of these semi-organized cancerous Cells and that of the simple Fungi, which are not unfrequently generated in the interior of living animal and vegetable structures, he proceeds to observe: "There can be little doubt that a cancerous tumor of any size may be developed from a single cell; and it seems very difficult to draw the line which shall separate such independent growths, on the one hand, from the ordinary tissues of the body (in their primordial condition), and, on the other, from structures really parasitic. It is interesting to remark that blood-vessels cannot be traced in these morbid productions at an early period of their formation, but that they make their appearance, as in the normal development of the tissues, at a later date; so that the disordered tendency cannot originate in the walls of the vessels (as some have thought), but must commence in the blood itself."

Dr. Klencke of Brunswick has recently published a work, with the view of shewing that the contagious character of many diseases is owing to the direct transmission of morbific *cells*—which seem to possess a semi-individual life—from one person or animal to another. After alluding to the opinion of Müller and Langenbeck that *Cancer* is communicable by the inoculation of carcinomatous matter, he mentions that he has succeeded, in a similar manner, in proving the contagiousness of *Melanosis*. The experiment was performed on a horse; some of the cellules, found floating in the black pulpy mass of a melanotic tumor, were inserted beneath the skin; and the result was the production of a growth similar to the original one. Dr. K. says, that various *Condylomatous tumors* also, as well as *Ozœna, Coryza*, &c. are transmissible in this way; and he goes so far as to state, that "the cellules of a recent *Coryza* are very different from the confervæ of an *Ozœna*, and that, as the disease declines, the cellules gradually disappear and become replaced by sporules of Confervæ!" He mentions, too, that he has detected the morbific cells of *Malignant Carbuncle* in the yellow-coloured discharge that flows from the gangrenous sores; and those of *Hydrophobia*, not only in the salivary glands of dogs affected with rabies, but also in a wound caused by their bite. The proximate cause of *Vaccinia* and *Variola* is, according to his researches, the existence of morbid Cells in the circulating fluid; and the severity of these diseases is, in general, proportionate to the number of the cells that are developed. Probably not a little fanciful conjecture is mixed up with these speculations of the learned doctor; but their simple announcement will be of use, by directing the attention of pathological microscopists to the changes, alike of the fluids and the solids, in various diseases.

Lastly, we may mention that the formation of *Tubercles* has been supposed to be dependent upon some lesion, or defect, in the structural transformation of the cells of the affected tissue. From the general debility of the nutrient powers, the normal cells are (it is imagined) only imperfectly converted into the parenchyma or proper substance of the part where the tubercles are developed; and thus, instead of forming a constituent portion of its organisation, they act as so many foreign and irritating bodies disseminated through it. Their appearance under the microscope gives considerable weight to this view of their origin; for, when examined with a moderate magnifying power, they are found to consist of half-formed *cells*, fibres, &c. associated with a granular matter that resembles coagulated albumen. If this pathological suggestion be correct, how much must depend on a judicious invigoration of the nutrient and reparative energies of the system, if we hope to arrest the progress of this fatal malady!

With this remark, we draw the present article to a close. All that now remains to do is to notice the several works, which we have brought under review.

Mr. *Owen*'s lectures present a minute and, we need scarcely say, a faithful description of the Anatomy of the various families of the great Invertebrate class of animals. A few physiological remarks are introduced here and there; but certainly not so frequently as the reader might desire. The present, the fifth, Edition of Dr. *Quain*'s Anatomy has already been deservedly stamped with very marked public approbation. The introductory chapters, by Dr. *Sharpey*, contain a well-written summary of the Cell-formation doctrine, as applied to human Anatomy. A more elaborate account of the same subject will be found in the work of Dr. *Todd* and Mr. *Bowman*; but unquestionably by far the ablest exposition of all its most interesting and instructive details is that given by Dr. *Carpenter* in his Popular Cyclopædia. This is a work of uncommon merit, and ought to be very widely known. Medical men will do well to read it first attentively themselves, and then recommend it to the perusal of all their *lay* friends that are persons of education and intelligence. The various descriptions are remarkably clear and graphic;—and the wood-cuts are among the very best which we have ever seen. Altogether, the publishers, as well as the talented author, deserve much praise for this beautiful volume, and we trust that the extent of its sale will testify the public's approval of their meritorious exertions.

